# Modulation of the Inflammatory Response by Adenovirus 36 in Patients with Obesity and Type 2 Diabetes: A Nested Case-Control Study Within a Cohort

**DOI:** 10.3390/v17040552

**Published:** 2025-04-10

**Authors:** Itzae Adonai Gutiérrez-Hurtado, Erika Martínez-López, Manuel Alejandro Rico-Méndez, Karla Mayela Bravo-Villagra, Héctor Eduardo Mendoza-Jaramillo, María del Pilar Sánchez-Rolón, Alejandra Betancourt-Núñez, Martha Patricia Gallegos-Arreola, José Carlos Tapia-Rivera, Andres López-Quintero

**Affiliations:** 1 Departamento de Biología Molecular y Genómica, Centro Universitario de Ciencias de la Salud, Universidad de Guadalajara, Guadalajara 44340, Mexico; itzae.gutierrez@academicos.udg.mx (I.A.G.-H.); erika.martinez@academicos.udg.mx (E.M.-L.); 2 Instituto de Nutrigenética y Nutrigenómica Traslacional, Centro Universitario de Ciencias de la Salud, Universidad de Guadalajara, Guadalajara 44340, Mexico; 3 Doctorado en Genética Humana, Centro Universitario de Ciencias de la Salud, Universidad de Guadalajara, Guadalajara 44340, Mexico; manuel.rico8557@alumnos.udg.mx (M.A.R.-M.); karla.bravo2318@alumnos.udg.mx (K.M.B.-V.); 4 Departamento de Ciencias Básicas para la Salud, Centro Universitario del Sur, Universidad de Guadalajara, Ciudad Guzmán 49000, Mexico; mendoza.hctor@gmail.com (H.E.M.-J.); sanchezrolon30@hotmail.com (M.d.P.S.-R.); 5 Departamento de Disciplinas Filosófico, Metodológico e Instrumentales, Centro Universitario de Ciencias de la Salud, Universidad de Guadalajara, Guadalajara 44340, Mexico; alejandra.bnunez@academicos.udg.mx; 6 División de Genética, Centro de Investigación Biomédica de Occidente (CIBO), Centro Médico Nacional de Occidente (CMNO), Instituto Mexicano del Seguro Social (IMSS), Guadalajara 44340, Mexico; marthapatriciagallegos08@gmail.com

**Keywords:** adenovirus 36, type 2 diabetes, obesity, IL-6, inflammatory response

## Abstract

Human adenovirus 36 (HAdV-36) is associated with obesity, potentially by promoting adipocyte proliferation and differentiation. Although linked to increased fat storage, HAdV-36 is also correlated with improved insulin sensitivity. Given its potential role in modulating adipose tissue and promoting a less inflammatory metabolic profile, its impacts on pro- and anti-inflammatory cytokine secretion remain unclear. Methods: This nested case-control study compared cytokine levels (IL-10, IL-2, IL-6, IL-8, and TNF-α) between patients with and without HAdV-36 infection. A total of 76 participants were included, with 37 in the control group (HAdV-36 negative) and 39 classified as cases (HAdV-36 positive). Results: HAdV-36 seropositive individuals exhibited significantly lower IL-6 levels and higher IL-8 levels than seronegative participants. Additionally, they had lower glucose levels, suggesting a potential link between HAdV-36 and metabolic regulation. Conclusions: These findings support the hypothesis that HAdV-36 may influence inflammatory and metabolic responses by modulating cytokine expression and glucose levels. Further research is needed to clarify the underlying mechanisms and their implications for metabolic health.

## 1. Introduction

The hypothesis of an infectious component in the development of obesity emerged in the 1990s with the discovery that the avian adenovirus SMAM-1 was associated with excessive intra-abdominal fat accumulation in chickens, paradoxically accompanied by low serum cholesterol and triglyceride levels [1]. Years later, it was identified that human adenovirus 36 (HAdV-36), a virus capable of infecting humans, similarly to SMAM-1, induced an increase in adipose tissue in chickens, mice, and male Rhesus monkeys [2,3].

HAdV-36 was observed to persist in the adipose tissue of infected animals for up to 16 weeks. However, despite the temporary presence of the virus in the tissues, its metabolic effects appear to be long-lasting. Additionally, it has been confirmed that the virus can be transmitted horizontally, suggesting a potential impact on the spread of its metabolic effects within a population [2,4,5].

Currently, various non-experimental studies in humans have documented an association between HAdV-36 and obesity. This association appears to be supported by the virus’s ability to promote adipocyte proliferation and differentiation. It has been observed that infection with this virus induces an increase in the expression of key factors involved in adipogenesis [6,7,8]. While being overweight and obese increases the risk of developing type 2 diabetes, insulin resistance, and dyslipidemia [9,10,11], HAdV-36 has demonstrated a paradoxical effect. Although it has been associated with greater fat storage and obesity, it has also been linked to improved insulin sensitivity, a lower incidence of type 2 diabetes, and a more favorable lipid profile in both humans and animal models [12,13,14].

Although the exact molecular basis by which HAdV-36 modulates glucose and lipid metabolism is not yet fully understood, a recent study suggested that this virus promotes adipogenesis by enhancing PPARγ expression and maintaining an adipogenic state over the long term. This effect appears to be mediated through the regulation of the GAS5 (growth arrest-specific transcript 5)/miR-18a axis, where reduced PPARγ levels in HAdV-36 seropositive individuals correlate with increased PPARγ expression, thereby facilitating adipocyte differentiation and proliferation [15,16].

**Figure 1:**
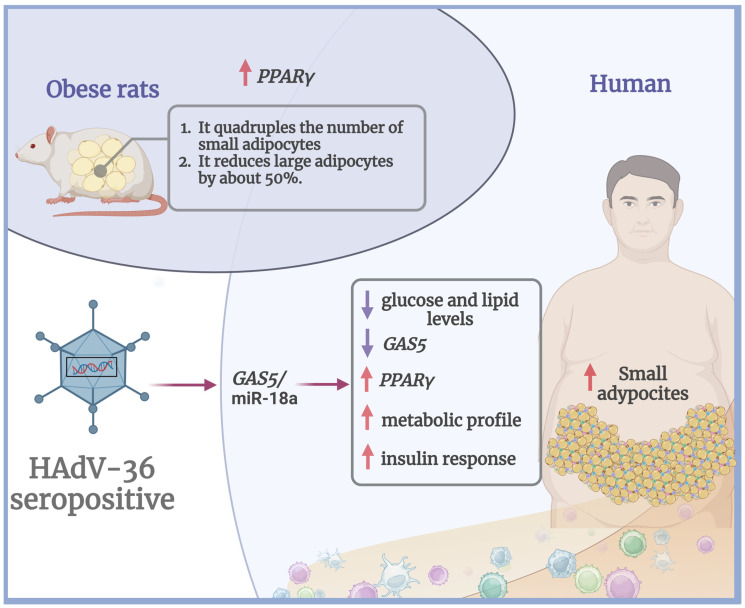
Impact of HAdV-36 seropositivity on adipogenesis and metabolic profile. In obese rats, increased PPARγ expression leads to a fourfold increase in the number of small adipocytes and a reduction in the number of large adipocytes by approximately 50%. In humans, HAdV-36 seropositivity is associated with lower GAS5 expression, increased PPARγ levels, and a shift toward a metabolic profile characterized by reduced glucose and lipid levels, improved insulin response, and predominance of small adipocytes.

An increase in PPARγ activation has been observed to quadruple the number of small adipocytes in the retroperitoneal and subcutaneous adipose tissues of obese rats, while reducing the number of large adipocytes by approximately 50% [17]. In humans, subcutaneous adipose tissue with a predominance of smaller adipocytes exhibits higher expression of lipogenic genes and increased insulin sensitivity [18]. Additionally, the secretion of pro-inflammatory cytokines, such as IL-6 and IL-8, has been shown to increase with adipocyte size, indicating that larger adipocytes are more strongly associated with a chronic inflammatory state. In contrast, smaller adipocytes appear to contribute to a more favorable metabolic profile characterized by lower inflammation and improved insulin response [19]. This mechanism could explain why the proliferation of small adipocytes in HAdV-36 seropositive individuals may be linked to reduced glucose and lipid levels, despite the virus’s association with increased adiposity (Figure 1).

Since HAdV-36 has been linked to adipose tissue modulation, potentially promoting the proliferation of small adipocytes and a less inflammatory metabolic profile, studying its impact on the secretion of pro- and anti-inflammatory cytokines is of great relevance, as it remains incompletely understood. In this context, the present study aims to compare IL-10, IL-2, IL-6, IL-8, and TNF-α levels between patients with and without HAdV-36 infection.

## 2. Materials and Methods

### 2.1. Study Design

This study is a nested case-control study within a cohort, based on participants from the study “Effect of Human Adenovirus 36 on Response to Metformin Monotherapy in Obese Mexican Patients with Type 2 Diabetes: A Prospective Cohort Study” [20].

### 2.2. Participants

All 103 volunteers from the original study were invited to participate; however, only 76 (26 men and 50 women) agreed to participate in the second investigation. All participants were referred from the Unidad Metabólica de Atención Nutricional Especializada (UMANE) in Ciudad Guzmán, Jalisco, Mexico.

To be included in the study, volunteers had to be over 18 years old, have obesity (BMI ≥ 30 kg/m^2^), and have been recently diagnosed with type 2 diabetes mellitus, according to the criteria established by the American Diabetes Association (ADA) [21]. Of the 76 participants, 37 were assigned to the control group (HAdV-36 negative), and 39 were classified as cases (HAdV-36 positive). The study was conducted between 1 November 2019 and 31 July 2020. The study was conducted between 1 November 2019 and 31 July 2020.

### 2.3. Biochemical Analysis

Blood samples were collected from the volunteers after a minimum of eight hours of fasting to analyze HAdV-36 serology and assess glycemic and lipid profiles. Blood extraction was performed by venipuncture in the forearm using a vacuum collection system.

The serum used for the qualitative identification of HAdV-36 antibodies was stored at −20 °C until analysis. The detection of antibodies against HAdV-36 was performed using an enzyme-linked immunosorbent assay (ELISA) kit (AdV36-Ab, MyBioSource^®^, San Diego, CA, USA, Kit No. MBS705802) following the manufacturer’s instructions.

For serological analysis of HAdV-36, blood samples were placed in 5 mL additive-free tubes and centrifuged at 3000 RPM for 10 min immediately after collection. The serum was then stored at −20 °C until processing. Detection of antibodies against the virus was performed using an ELISA assay with the HAdV-36 ELISA antibody kit (AdV36-Ab, MyBioSource, San Diego, CA, USA, catalog No. MBS705802) in conjunction with a Biotek Synergy TH multimode microplate reader (Winooski, VT, USA) calibrated to a wavelength of 450 nm. The optical density of each sample was determined according to the manufacturer’s specifications, with samples considered positive if their optical density ratio was ≥2.1 compared to that of the negative control. The intra-assay precision reported by the manufacturer indicated a variability of less than 15% within and between the tests.

Glucose and lipid profile analyses were performed using a semi-automated clinical chemistry analyzer (Spinlab, Spinreact, Sant Esteve de Bas, Spain). To determine the biochemical parameters, commercial reagent kits were used, including the hexokinase glucose assay (ref: 1001201), cholesterol CHOD-POD assay (ref: 41021), and triglyceride GPO-POD assay (ref: 1001313), all from Spinreact. The analyses were conducted at the Clinical Analysis Laboratory of Centro Universitario del Sur, University of Guadalajara.

Serum levels of IL-6, IL-8, IL-10, and TNF-α were quantified using a sandwich enzyme-linked immunosorbent assay (Sandwich ELISA). The Bio-Plex Pro Human Cytokine Panel 8-plex assay kit (BIO-RAD, catalog No. M50000007A) was used in the Bio-Plex Pro™ Assay System, Hercules, CA, USA (BIO-RAD). Readings were obtained using a Bio-Plex 200 microbead reader (BIO-RAD), which was calibrated and operated according to the manufacturer’s specifications. All analytical procedures were carried out at the University of Guadalajara.

### 2.4. Anthropometric Assessment

Anthropometric measurements were conducted using bioelectrical impedance analysis (BIA) with a Tanita Ironman BC-558 body composition monitor. Height was determined using a Seca 206 wall-mounted stadiometer, manufactured by Seca GmbH & Co. KG, based in Hamburg, Germany. Waist circumference was measured using a Lufkin W606PMMX metal tape measure, manufactured by Apex Tool Group, LLC (Crescent Lufkin), based in Sparks, Maryland, United States.. BMI was calculated as weight (kg) divided by height (m) squared to classify participants with obesity.

### 2.5. Ethical Considerations

This study was conducted in accordance with the Ethical Principles for Medical Research Involving Human Subjects established in the Declaration of Helsinki (2013 version) and the International Ethical Guidelines for Biomedical Research Involving Human Subjects by CIOMS. The research was approved by the Ethics Committee f the Centro Universitario del Sur, University of Guadalajara, under approval code CI/T/009/19. All participants provided written informed consent prior to inclusion in the study.

### 2.6. Statistical Analysis

A descriptive analysis of the data was performed. Quantitative variables are presented as the median and 25th and 75th percentiles, as their distribution did not meet the assumptions of normality according to the Kolmogorov-Smirnov/Shapiro-Wilk test. Qualitative variables are expressed as frequencies and percentages.

Cytokine levels between individuals with and without human adenovirus 36 (HAdV-36) were compared using the Mann−Whitney U test. Qualitative variables were compared using the chi-square (χ^2^) test.

A linear regression analysis was conducted to assess the association between the interleukin levels and the presence of HAdV-36. The β coefficient is presented with its corresponding 95% confidence interval. Both crude and adjusted values (adjusted for age and sex) are reported.

Additionally, hierarchical cluster analysis was performed using IL-10, IL-2, IL-6, IL-8, and TNF-α, dichotomized at the 50th percentile. Ward’s method was applied with the squared Euclidean distance as the clustering criterion.

Statistical significance was set at *p* < 0.05. Data analysis was conducted using R software version 4.1.2.

## 3. Results

A total of 76 participants were included in this study, of whom 26 (34.2%) were men and 50 (65.8%) were women. Among them, 37 were assigned to the control group (HAdV-36 negative) and 39 to the case group (HAdV-36 positive). In the control group, 11 were men and 26 were women, whereas in the case group, 15 were men and 24 were women. No significant differences were observed in the prevalence of the virus according to sex.

No significant differences were observed in age, height, weight, or BMI between the patients with and without HAdV-36. Similarly, no differences were observed in body fat percentage, anthropometric measurements (waist circumference, hip circumference, and waist-to-hip ratio), or total cholesterol and triglyceride levels.

However, statistically significant differences were found in serum glucose levels, which were lower in HAdV-36 seropositive individuals, suggesting a potential improvement in insulin sensitivity in this group.

Table 1 presents a comparison of the anthropometric and metabolic characteristics of patients with and without HAdV-36 infection.

Serum levels of IL-10, IL-2, IL-6, IL-8, and TNF-α were analyzed and compared between patients with and without HAdV-36 infection. It was found that IL-6 levels were significantly lower in the HAdV-36 positive group (0.39 pg/mL vs. 0.78 pg/mL, *p* = 0.001), while IL-8 levels were significantly higher in these patients (12.60 pg/mL vs. 5.40 pg/mL, *p* = 0.002). No statistically significant differences were observed in IL-10, IL-2, or TNF-α levels between the two groups. The detailed results are presented in Table 2.

A linear regression analysis was performed to evaluate the association between HAdV-36 infection and levels of IL-10, IL-2, IL-6, IL-8, and TNF-α. The results identified a significant positive association between HAdV-36 infection and elevated IL-8 levels in the adjusted model (adjusted β = 45.03, 95% CI: 0.80, 89.27, *p* < 0.05). In the crude model, the association did not reach statistical significance (crude β = 41.21, 95% CI: −1.84, 84.25).

Conversely, the analysis of IL-10, IL-2, IL-6, and TNF-α did not reveal any statistically significant associations in either model. The results are presented in Table 3.

A hierarchical cluster analysis using squared Euclidean distance was performed to classify the participants into two groups based on IL-10, IL-2, IL-6, IL-8, and TNF-α levels. Cluster 1 was characterized by low levels of IL-10, IL-2, IL-8, and TNF-α, while Cluster 2 exhibited elevated levels of these cytokines. In contrast, IL-6 levels did not differ significantly between clusters (*p* = 0.781), suggesting that its variability did not contribute to the formation of the groups. The characteristics of these clusters are presented in Table 4.

A Chi-square (χ^2^) analysis was performed to assess whether HAdV-36 infection was associated with any of the clusters identified using hierarchical cluster analysis. In Cluster 1, 57.1% of the participants were negative for HAdV-36, while 42.9% were positive. In Cluster 2, 41.7% were negative and 58.3% were positive for HAdV-36. Statistical analysis revealed no significant difference in the distribution of HAdV-36 between the clusters (*p* = 0.404). The results are presented in Table 5.

Finally, anthropometric and metabolic characteristics were compared between participants grouped into two clusters, defined using hierarchical cluster analysis. No statistically significant differences (*p* > 0.05) were found in any of the analyzed variables, indicating that both clusters exhibited similar characteristics in terms of age, weight, BMI, body composition, anthropometric measurements, and metabolic profile. Although Cluster 1 showed slightly higher values for weight (96.3 kg vs. 91.1 kg), BMI (35.3 vs. 34.3), and waist circumference (104.5 cm vs. 100.0 cm), these differences were not statistically significant. The detailed results are presented in Table 6.

## 4. Discussion and Conclusions

In this study, no association was found between age, height, weight, BMI, and the presence of HAdV-36. This is primarily due to the selection criteria for participants, as the case-control study design aimed to ensure that the comparison groups were as similar as possible, differing only in the presence or absence of the virus. This approach allowed for a more precise evaluation of the measured cytokines, minimizing the influence of other variables on the results.

Among the statistically significant findings, HAdV-36 seropositive participants exhibited lower glucose levels than seronegative individuals. The relationship between HAdV-36 and glucose regulation has been extensively studied in animal models, in vitro studies, and human subjects [22,23,24]. Previous studies conducted in the Mexican population have reported that seropositivity for HAdV-36 is associated with reduced blood glucose levels in individuals with obesity [12]. Similarly, a study in Sweden found that HAdV-36 infection was associated with a lower incidence of type 2 diabetes and improved insulin sensitivity in adults [25].

The molecular mechanisms by which HAdV-36 improves glucose levels remain poorly understood. However, several well-supported theories exist, one of the most widely accepted attributing this effect to the E4orf1 protein. E4orf1 is a 125-amino-acid protein encoded by the E4 open reading frame 1 (E4orf1) gene of human adenoviruses, which has been shown to enhance glucose uptake in preadipocytes, adipocytes, and myoblasts while reducing glucose production in hepatocytes. Additionally, it prevents hepatic lipid accumulation, thereby slowing the progression of hepatic steatosis in animal models. Experimental studies have also demonstrated that E4orf1 improves glycemic control and reduces the need for endogenous insulin to regulate glucose levels [24,26,27,28]. 

E4orf1 physically interacts with MYC, which is a key factor in adenovirus replication. This interaction not only promotes nucleotide biosynthesis from glucose metabolites—facilitating viral replication—but also directly influences glucose metabolism in the host cell. Through MYC activation, E4orf1 enhances glycolysis by upregulating the transcription of glycolytic genes, suggesting a direct mechanism by which HAdV-36 modulates glucose utilization in the body [29].

A recent study suggested that the adipogenic effect of HAdV-36 may be mediated by the regulation of long non-coding RNAs (lncRNAs) involved in adipocyte differentiation. Specifically, HAdV-36 infection has been shown to downregulate GAS5, an anti-adipogenic lncRNA that has also been identified as a tumor suppressor gene. The reduction in GAS5 is associated with the increased expression of key adipogenic factors, such as PPAR-γ, C/EBP-α, and FABP4, promoting adipocyte proliferation and differentiation. These findings suggest that HAdV-36 enhances adipogenesis by downregulating GAS5, thereby eliminating its inhibitory effect on adipocyte differentiation [15,30].

The decrease in GAS5 leads to an upregulation of PPAR-γ, which has been previously reported to be a key mechanism in the regulation of energy metabolism. Since PPAR-γ plays a central role in improving insulin sensitivity, this mechanism could explain the lower glucose levels observed in HAdV-36 seropositive individuals, as well as the increase in adiposity [31,32,33].

Regarding cytokine levels, IL-6 was significantly lower in the HAdV-36 positive group, whereas IL-8 was significantly higher in these patients. However, no differences were observed in the levels of other cytokines analyzed between the study groups.

IL-6 is a pro-inflammatory cytokine primarily secreted by M1 macrophages and has been extensively studied for its role in chronic inflammatory processes and its association with metabolic diseases, such as type 2 diabetes. Previous studies have reported that fibroblasts produce IL-6 in response to elevated glucose levels, making it one of the primary cytokines released in a hyperglycemic environment [34,35,36,37]. In this context, the lower glucose levels observed in HAdV-36-positive participants in the present study could explain the lower IL-6 levels in this group.

In contrast to the findings of the present study, some investigations have reported higher levels of IL-6 and other cytokines (IL-1β, IL-10, and IP-10) in HAdV-36 seropositive patients [35,38]. This difference could be explained by the methodology used in those studies, where participants were classified according to BMI into categories of overweight, obesity, and severe obesity. In these investigations, higher cytokine levels were observed in HAdV-36-positive individuals, particularly among adolescents with severe obesity, a condition known to be associated with a heightened inflammatory state [35]. In contrast, our study did not include participants with severe obesity; moreover, no significant differences in BMI were found between the HAdV-36 positive and negative groups. The median BMI was slightly lower in seropositive individuals (31.2) than in seronegative individuals (34). These differences may help explain the divergent cytokine profiles reported in previous studies. Additionally, our results indicate that the main variable associated with changes in IL-6 levels among HAdV-36 positive participants was glucose, suggesting a possible alternative mechanism of inflammatory regulation in these individuals. One of the key findings of this study was that IL-8 levels were significantly higher in the HAdV-36 positive group compared to the negative group. This result contrasts with that of previous studies, in which no significant association was found between IL-8 levels and HAdV-36 seropositivity [35].

A possible explanation for this difference lies in the characteristics of the study population, which consisted of patients with type 2 diabetes who were undergoing dietary and pharmacological treatment for glucose control. It has been reported that plasma levels of IL-6 and IL-8 are elevated in individuals with obesity [39]. However, it has also been documented that, following weight loss, IL-6 levels tend to decrease, while IL-8 levels remain elevated [40]. Therefore, it is possible that, as a result of the intervention, weight loss and improved glucose metabolism influenced the reduction in IL-6 levels but did not have the same effect on IL-8 levels.

Another possible explanation for the observed IL-8 levels is their relationship with physical activity. Previous studies have reported a significant reduction of up to 35% in IL-8 concentrations after the first six weeks of intervention in groups that engaged in aerobic training. However, no tool was used to assess physical activity levels in the present study. Therefore, it is possible that this variable may have influenced the differences observed in the results [40]. Additionally, a cluster analysis was conducted in which the cytokine levels were dichotomized. This analysis allowed us to classify the participants into two clusters to identify distinct cytokine expression patterns. However, none of these analyses revealed any statistically significant differences between the study groups.

The cluster analysis results suggest that in the studied population, the presence of HAdV-36 was not associated with significant changes in the analyzed cytokine levels. The absence of differences between clusters also suggests that other factors may modulate the inflammatory response in these individuals.

This study has some limitations that should be acknowledged. First, the exclusive use of IgG serology (via ELISA) to determine prior exposure to HAdV-36 may limit the precision of the viral exposure classification. Although ELISA assays offer practical advantages in terms of speed and scalability, their diagnostic performance, especially specificity, can vary among commercial kits. In our case, the assay was validated for research purposes and applied according to the manufacturer’s criteria; however, virus neutralization assays, which are considered the gold standard, were not feasible within the scope of this study.

Second, cytokine detection was unsuccessful in 24 participants, potentially due to sample degradation, matrix effects, or cytokine levels below the assay detection threshold. This limitation may have impacted the interpretation of the inflammatory profiles and reduced the generalizability of the findings.

Our findings suggest that HAdV-36 seropositivity is associated with lower blood glucose levels and alterations in cytokine expression, characterized by reduced IL-6 and increased IL-8 levels. These results reinforce the hypothesis that HAdV-36 may play a role in the regulation of glucose metabolism and modulation of inflammatory responses.

However, to better understand the underlying mechanisms, future studies should incorporate larger sample sizes and longitudinal designs to evaluate the temporal dynamics of cytokine expression and its relationship with viral seropositivity. This would contribute to more robust evidence of the impact of HAdV-36 on metabolic and inflammatory regulation, as well as its potential implications in metabolic diseases.

## Figures and Tables

**Table 1:** Anthropometric and metabolic characteristics of patients with and without HAdV-36 infection. Data are expressed as the median and 25th and 75th percentiles. Median differences were analyzed using the Mann−Whitney U test. *p* value < 0.05.

				HAdV-36						
	Total (*n* = 76)			Negative (*n* = 37)			Positive (*n* = 39)			*p*
	Median	25th Percentile	75th Percentile	Median	25th Percentile	75th Percentile	Median	25th Percentile	75th Percentile	
Age (years)	49.5	39.0	55.0	51.0	44.0	58.0	48.0	37.0	55.0	0.154
Height (cm)	162.0	156.0	170.0	159.0	154.0	165.0	165.0	157.0	171.0	0.117
Weight (kg)	92.3	83.2	100.4	89.6	82.7	96.5	95.3	83.9	101.7	0.308
BMI (kg/m^2^)	34.5	31.4	38.4	34.0	31.8	38.7	34.5	31.2	38.2	0.743
Body fat (%)	35.2	31.7	40.1	35.5	32.9	40.2	33.0	30.2	40.0	0.266
Waist (cm)	102.0	95.5	114.5	102.0	94.0	114.0	103.0	96.0	117.0	0.336
Hip (cm)	103.0	92.5	120.0	101.0	92.0	113.0	104.5	93.0	121.0	0.436
Waist-to-hip ratio	1.0	0.9	1.1	1.0	0.9	1.1	1.0	0.9	1.1	0.666
Glucose (mg/dL)	114.0	93.0	162.0	123.0	111.0	186.0	100.0	89.0	138.0	0.007 *
Cholesterol (mg/dL)	172.0	144.0	195.0	177.2	144.0	195.0	171.0	144.0	195.0	0.938
Triglycerides (mg/dL)	175.0	112.9	234.5	169.2	95.0	235.0	181.0	122.0	234.0	0.647

* *p* < 0.05, statistically significant difference.

**Table 2:** Inflammatory markers in patients with and without HAdV-36 infection. Corrected inflammatory score, adjusted by subtracting IL-10, based on IL-2, IL-6, IL-8, and TNF-α (P50). Quantitative variables are expressed as medians and 25th and 75th percentiles. Group differences were analyzed using the Mann-Whitney U test. *p* value < 0.05.

	HAdV-36		
	Negative (*n* = 26, 50%)	Positive (*n* = 26, 50%)	*p*
IL-10 (pg/mL)	0.54 (0.32, 2.17)	0.74 (0.32, 0.99)	0.825
IL-2 (pg/mL)	0.97 (0.81, 2.41)	0.93 (0.71, 1.12)	0.273
IL-6 (pg/mL)	0.85 (0.61, 1.21)	0.39 (0.23, 0.78)	0.001 *
IL-8 (pg/mL)	5.36 (2.92, 9.11)	17.30 (5.57, 53.37)	0.002 *
TNF-α (pg/mL)	5.53 (4.25, 6.99)	6.31 (4.70, 7.09)	0.441
Inflammatory score based on IL-2, IL-6, IL-8, TNF-α (P50)	6.00 (5.00, 7.00)	6.00 (5.00, 7.00)	0.977
Corrected inflammatory score (P50)	5.00 (4.00, 5.00)	4.00 (4.00, 5.25)	0.788

* *p* < 0.05, statistically significant difference.

**Table 3:** Association between HAdV-36 infection and inflammatory markers.

	Unadjusted Model		Adjusted Model	
	β	95% CI	β	95% CI
IL-10	4.50	−4.78, 13.79	3.27	−6.23, 12.78
IL-2	−0.20	−0.79, 0.38	−0.19	−0.80, 0.42
IL-6	−1.97	−5.08, 1.13	−1.81	−4.99, 1.36
IL-8	41.21	−1.84, 84.25	45.03	0.80, 89.27 *
TNF-α	−0.19	−1.24, 0.86	0.03	−0.99, 1.04

* *p* < 0.05, statistically significant difference.

**Table 4:** Hierarchical cluster analysis based on the inflammatory markers. Interleukins were dichotomized at the 50th percentile. The clusters included only participants with complete measurements for all the interleukins of interest. Differences between qualitative variables were analyzed using the chi-square (χ^2^) test. Statistical significance was set at *p* < 0.05.

		Squared Euclidean Distance				
		Cluster 1 (*n* = 28)		Cluster 2 (*n* = 24)		*p*
		n	%	n	%	
IL-10 (pg/mL)	≤0.54	23	82.1	3	12.5	<0.001 *
	≥0.55	5	17.9	21	87.5	
IL-2 (pg/mL)	≤0.81	20	71.4	2	8.3	<0.001 *
	≥0.82	8	28.6	22	91.7	
IL-6 (pg/mL)	≤0.61	13	46.4	13	54.2	0.781
	≥0.62	15	53.6	11	45.8	
IL-8 (pg/mL)	≤7.14	20	71.4	6	25.0	0.001 *
	≥7.15	8	28.6	18	75.0	
TNF-α (pg/mL)	≤5.53	19	67.9	4	16.7	<0.001 *
	≥5.54	9	32.1	20	83.3	

* *p* < 0.05, statistically significant difference.

**Table 5:** Association analysis of HAdV-36 infection by cluster. Data are presented as frequency and percentage.

		Squared Euclidean Distance, 2 Cat				
		Cluster 1		Cluster 2		*p*
		*n*	%	*n*	%	
HAdV-36	Negative	16	57.1	10	41.7	0.404
	Positive	12	42.9	14	58.3	

**Table 6:** Comparison of anthropometric and metabolic characteristics by cluster. Data are expressed as medians and 25th and 75th percentiles, and group differences were analyzed using the Mann-Whitney U test. *p* < 0.05.

	Squared Euclidean Distance						
	Cluster 1			Cluster 2			
	Median	25th Percentile	75th Percentile	Median	25th Percentile	75th Percentile	*p*
Age (years)	51.0	43.0	58.5	49.0	46.5	55.0	0.639
Height (cm)	162.0	159.0	175.5	161.0	155.5	167.5	0.177
Weight (kg)	96.3	88.3	107.4	91.1	80.7	98.3	0.074
BMI (kg/m^2^)	35.3	31.5	39.5	34.3	31.6	36.6	0.485
Body fat (%)	35.2	32.2	41.5	37.7	32.1	43.9	0.576
Waist (cm)	104.5	94.5	117.5	100.0	97.0	111.0	0.514
Hip (cm)	107.0	96.3	123.5	100.5	87.8	116.5	0.195
Waist-to-hip ratio	1.0	0.9	1.1	1.0	0.9	1.1	0.368
Glucose (mg/dL)	99.8	86.4	170.6	112.0	99.5	149.5	0.326
Cholesterol (mg/dL)	175.1	135.5	204.4	167.5	144.0	199.0	0.601
Triglycerides (mg/dL)	170.6	114.0	254.5	184.5	102.5	241.0	0.847

## Data Availability

The data presented in this study are available upon request from the corresponding author. The data are not publicly available due to the confidentiality agreement with the study subjects.
